# Aerosol Delivery of a Candidate Universal Influenza Vaccine Reduces Viral Load in Pigs Challenged with Pandemic H1N1 Virus

**DOI:** 10.4049/jimmunol.1502632

**Published:** 2016-05-06

**Authors:** Sophie B. Morgan, Johanneke D. Hemmink, Emily Porter, Ross Harley, Holly Shelton, Mario Aramouni, Helen E. Everett, Sharon M. Brookes, Michael Bailey, Alain M. Townsend, Bryan Charleston, Elma Tchilian

**Affiliations:** * The Pirbright Institute, Surrey GU24 0NF, United Kingdom;; † School of Veterinary Sciences, University of Bristol, Bristol BS40 5DU, United Kingdom;; ‡ The Jenner Institute, University of Oxford, Oxford OX3 7DQ, United Kingdom;; § Virology Department, Animal and Plant Health Agency, Weybridge, Surrey KT15 3NB, United Kingdom; and; ¶ Weatherall Institute of Molecular Medicine, University of Oxford, Oxford OX3 9DS, United Kingdom

## Abstract

Influenza A viruses are a major health threat to livestock and humans, causing considerable mortality, morbidity, and economic loss. Current inactivated influenza vaccines are strain specific and new vaccines need to be produced at frequent intervals to combat newly arising influenza virus strains, so that a universal vaccine is highly desirable. We show that pandemic H1N1 influenza virus in which the hemagglutinin signal sequence has been suppressed (S-FLU), when administered to pigs by aerosol can induce CD4 and CD8 T cell immune responses in blood, bronchoalveolar lavage (BAL), and tracheobronchial lymph nodes. Neutralizing Ab was not produced. Detection of a BAL response correlated with a reduction in viral titer in nasal swabs and lungs, following challenge with H1N1 pandemic virus. Intratracheal immunization with a higher dose of a heterologous H5N1 S-FLU vaccine induced weaker BAL and stronger tracheobronchial lymph node responses and a lesser reduction in viral titer. We conclude that local cellular immune responses are important for protection against influenza A virus infection, that these can be most efficiently induced by aerosol immunization targeting the lower respiratory tract, and that S-FLU is a promising universal influenza vaccine candidate.

## Introduction

Influenza A virus (IAV) infection is a global health threat to livestock and humans, causing substantial mortality and morbidity. Pandemic and avian-like H1N1 and H3N2 IAV are endemic in pigs and humans in addition to H1N2 in pigs. The human pandemic 2009 H1N1 strain is also found in pigs, and human viruses or human-origin gene segments frequently adapt to transmit efficiently in pigs. Thus, pigs play a critical role in the emergence and epidemiology of novel IAV.

Apart from the economic loss to farmers attributable to IAV infections of pigs, the generation of new reassortant strains of IAV poses a zoonotic threat to humans. Immunization is a cost-effective control measure to combat influenza infection, but the rapid evolution of the virus is a major obstacle. Conventional inactivated IAV vaccines are strain specific and protection correlates with the presence of neutralizing Ab. However, IAV infection and live attenuated viral vaccines have been shown to induce a degree of cross-protection in several species (1), suggesting that it may be possible to develop an effective “universal vaccine” for use in pigs and humans.

Influenza virus in which the hemagglutinin (HA) signal sequence is suppressed (S-FLU) is a candidate universal vaccine. Suppression of the signal sequence limits expression of the viral encoded HA protein to the cytosol. The HA protein coating the S-FLU virus is provided from a transfected complementing cell line by pseudotyping (2), and is expressed from a codon-optimized cDNA lacking 5′ and 3′ untranslated regions of the viral RNA. This design allows infection by the vaccine virus to occur once only, resulting in attenuation. Replication of vaccine in the lung or nose is prevented, but all of the conserved viral proteins are expressed in the cytosol of S-FLU–infected cells for optimal Ag presentation (3).

Immunization with S-FLU initiates broadly reactive cell-mediated immune responses to conserved viral Ags shared by all influenza viruses, which limit viral replication in the lungs of mice and ferrets even when heterologous challenge IAV are used (2, 4). Neutralizing Ab is not induced.

In this study, we tested the effect of S-FLU–expressing pandemic origin H1 or H5 HA on viral load and pathology after challenge with IAV-expressing homologous or heterologous HA in pigs.

## Materials and Methods

### S-FLU vaccine and influenza challenge virus

The design and production of S-FLU, that is, [S-eGFP/N1(Eng)].H1(Eng), have been described previously (2). We used two vaccines, H1 and H5 S-FLU. H1 S-FLU expresses the HA and neuraminidase (NA) of the A/England/195/2009(pandemic [pdm]H1N1) (N1 http://www.ncbi.nlm.nih.gov/nuccore/GQ166659 and surface H1 HA http://www.ncbi.nlm.nih.gov/nuccore/237689564) and internal protein genes of PR8. H5 S-FLU expresses the HA of the highly pathogenic avian influenza virus (HPAIV) A/Vietnam/1203/2004 (H5N1 clade 1, VN/04) with the NA and internal protein genes of PR8 (4). The H5 HA cDNA was codon optimized and the polybasic site was replaced with a trypsin cleavage site.

The pig isolate of A/Swine/England/1353/09(pdmH1N1) (A/Sw/Eng/1353/09) was provided by Dr. Sharon Brookes (Animal and Plant Health Agency, U.K.). Virus stocks were propagated in the allantoic cavities of 9- to 11-d-old embryonated specific pathogen-free hens’ eggs and used for viral challenge. For all serological and immunological assays the virus was propagated in Madin–Darby canine kidney (MDCK) cells.

### Animals and immunization

All experiments were approved by the ethical review processes at the Pirbright Institute and the University of Bristol, according to the U.K. Animal (Scientific Procedures) Act 1986.

Twenty-four twelve-week-old Landrace cross female pigs were obtained from a commercial high health status herd. Pigs were screened for absence of influenza A infection by matrix gene real-time RT-PCR and for Ab-free status hemagglutination inhibition using four swine influenza virus Ags. Pigs were randomly divided into four groups of six and immunized as follows: 1) control received 4 ml DMEM, 0.1% (w/v) BSA, and 1× penicillin and streptomycin intratracheally (i.t.), 2) H1 S-FLU was administered i.t. at 2 × 10^7^ tissue culture–infective dose (TCID)_50_ in 4 ml (H1 i.t.), 3) H1 S-FLU was administered by aerosol (aer) by delivering ∼6.85 × 10^6^ TCID_50_ in 1 ml (H1 aer), and 4) H5 S-FLU was administered i.t. at 8 × 10^7^ TCID_50_ in 4 ml (H5 i.t.). The animals received an identical booster immunization 28 d later. For i.t. immunization, following sedation, a 20-gauge needle was inserted through the skin and wall of the trachea cranial to the sternum and for aerosol immunization an InnoSpire Deluxe Philips Respironics nebulizer was fixed to a small-sized anesthetic mask held over the animal’s nose and mouth. The aerosol delivery process was designed to deliver 6.85 × 10^6^ TCID_50,_ although the actual retained dose in the lungs cannot be measured directly. However, we have determined that the nebulization process results in between 20 and 50% loss of viability of either S-FLU or the challenge virus, which means that the actual dose of viable S-FLU administered by aerosol is lower.

For logistical reasons, two influenza virus challenges were performed, with half of the animals challenged at 31 d postboost (dpb) and the remainder at 45 dpb. Pigs were rehoused so that each challenge pen contained one animal from each immunization group. Animals were challenged intranasally with 6 × 10^6^ 50% egg infectious dose, equivalent to 1.5 × 10^5^ PFU/pig of A/Sw/Eng/1353/09. Two milliliters was administered to each nostril using a mucosal atomization device (MAD300; Wolfe Tory Medical).

### Pathological and histopathological examination of lungs

Animals were humanely killed 3 d postchallenge (dpc) with an overdose of pentobarbital sodium anesthetic. The lungs were removed and digital images taken of the dorsal and ventral aspects. Macroscopic pathology scoring was performed blind at the University of Bristol using ImageJ v1.46r software (National Institutes of Health) to determine the percentage of the total surface area of the lung (dorsal and ventral aspects) affected by consolidation.

Lung tissue samples were collected into 10% neutral buffered formalin for routine histological processing (University of Bristol). Formalin-fixed tissues were paraffin wax embedded and 5-μm sections were cut and then stained with H&E.

Microscopic changes in the sections of lung were scored by a veterinary pathologist blinded to the treatment group. Five parameters were each scored on a 5-point scale of 0–4 and then summed to give a total slide score ranging from 0 to 20. Scoring criteria (Supplemental Table I) were based on a previously published method (5) with modifications. In cases where more than one lung sample was taken from an individual pig (e.g., lesion and nonlesion sites), the section with the highest composite score was used for subsequent statistical analysis. In these cases, this always corresponded to a sample containing a lung lesion that was visible on gross examination.

### Tissue sample processing

Two nasal swabs (one per nostril) were taken at 0, 1, 2, and 3 dpc. The swabs were placed into 2 ml virus transport medium comprising tissue culture medium 199 (Sigma-Aldrich) supplemented with 25 mM HEPES, 0.035% sodium bicarbonate, 0.5% BSA, penicillin, streptomycin, and nystatin, vortexed, centrifuged to remove debris, and stored at −80°C for subsequent virus titration.

Serum and citrate anticoagulated blood samples were collected at the start of the study (prior to immunization), at days 0, 7, 13, and 28 dpb and 0 and 3 dpc. Citrate blood samples were diluted 1:1 in PBS before density gradient centrifugation at 1200 × *g* for 30 min over Histopaque 1.083g/ml (Sigma-Aldrich). PBMCs were harvested from the interface, washed, and RBCs were lysed with ammonium chloride lysis buffer, washed again, and cryopreserved in FCS (Life Technologies) with 10% DMSO (Sigma-Aldrich).

Bronchoalveolar lavage (BAL) was collected from the entire right lung lobe with 150 ml virus transport medium (described above). BAL cells were isolated by centrifugation of the lavage fluid at 800 × *g* for 15 min. The supernatant was removed, aliquoted, and frozen for Ab analysis, whereas the cell pellet was washed in PBS and filtered through a 70-μm cell strainer and cryopreserved.

Tracheobronchial lymph nodes (TBLN) were dissected out. TBLN cells were dissociated into a single-cell suspension with the gentleMACS Octo dissociator (Miltenyi Biotec, Woking, U.K.), using C tubes (Miltenyi Biotec) with 3 ml RPMI 1640. The single-cell suspension was filtered twice using a 70-μm cell strainer, washed, and RBCs were lysed. Cells were washed again and cryopreserved.

The accessory lung lobes were dissected out and frozen at −80°C for subsequent virus titration. The whole lobe was homogenized using the gentleMACS Octo dissociator, the homogenate was clarified by centrifugation, and supernatant was used for virus titration.

### Virus titration

Viral titers in nasal swabs and the lung accessory lobe were determined by plaque assay on MDCK cells. Samples were 10-fold serially diluted and 100 μl was overlaid on confluent MDCK cells in 12-well tissue culture plates. After 1 h, the plates were washed and 2 ml 2% agarose/medium (1:3) was overlaid. Plates were incubated at 37°C for 48 h and plaques visualized using 0.1% crystal violet.

### Ab ELISA, HA inhibition, and microneutralization assay

Influenza-specific Ab titers in serum and BAL fluid were determined by HA inhibition (HAI) using standard protocols (6). Briefly, H1 HAI Ab titers were determined using 0.5% chicken RBCs and A/Sw/Eng/1353/09 at a concentration of 4 HA units/ml.

Neutralizaing Ab titers were determined in serum and BAL fluid using a microneutralization assay as described in the *WHO Manual on Animal and Influenza Diagnosis and Surveillance*. Briefly, samples were diluted 10-fold and heat inactivated at 56°C for 30 min, and further serially diluted 2-fold in PBS. Samples were then incubated with 4 × 10^4^ PFU/ml A/swine/England/1353/09(pdmH1N1) in serum-free DMEM for 1 h at 37°C in 5% CO_2_. The virus/sample mix was then added onto confluent MDCK cells in a 96-well plate and incubated at 37°C in 5% CO_2_. After 1 h, the plates were washed and overlaid with serum-free DMEM containing 2 μg/ml TPCK trypsin. Plates were incubated at 37°C for 48 h and cytopathic effect was visualized using 0.1% crystal violet. Titers were determined as the final dilution of serum that prevents cytopathic effect on MDCK cells following incubation with virus.

The IgG and IgA ELISAs were performed in 96-well ELISA plates (BD Biosciences) coated with a preoptimized concentration of A/swine/England/1353/09(pdmH1N1). Two-fold dilutions of BAL fluid samples were added starting from 1:2 dilution. Binding of Abs was detected using preoptimized concentrations of polyclonal goat-anti pig IgG HRP or IgA-HRP (AbD Serotec) and tetramethylbenzidine substrate (BioLegend). Ab values were expressed as endpoint titers defined as the highest dilution at which the OD was higher than twice the background OD.

### IFN-γ ELISPOT

Frequencies of IFN-γ secreting in PBMCs, BAL, and TBLN cells were determined by ELISPOT using either fresh or cryopreserved cells. MultiScreen-HA ELISPOT plates (Merck Millipore) were coated with 0.5 μg/ml anti-pig IFN-γ (clone P2G10; BD Pharmingen) in carbonate buffer and incubated at 4°C overnight. The plates were washed five times in PBS and blocked using 4% (w/v) milk powder in PBS for 2 h. After five washes in PBS, 5 × 10^5^ cells were seeded in triplicate wells and stimulated with either live MDCK cell–grown A/Sw/Eng/1353/09 (multiplicity of infection [MOI] of 6), medium control, or 10 μg/ml Con A (Sigma-Aldrich). Plates were incubated overnight or for 40 h at 37°C in a 5% CO_2_ incubator, depending on whether fresh or frozen cells were used, followed by washes with PBS, 0.05% Tween 20, and addition of 0.25 μg/ml anti-pig biotinylated IFN-γ detection Ab (clone P2C11; BD Pharmingen). Plates were incubated for 2 h at room temperature, washed five times, and streptavidin–alkaline phosphatase (Invitrogen) was added for a further 1 h at room temperature. Spots were visualized using an alkaline phosphatase substrate kit (Bio-Rad) and the reaction was stopped using tap water. Immunospots were counted using the AID ELISPOT reader (AID Autoimmun Diagnostika). Results were expressed as number of IFN-γ–producing cells per 10^6^ cells after subtraction of the average number of IFN-γ^+^ cells in medium control wells.

### Flow cytometry

Cryopreserved mononuclear cells from blood, TBLN, and BAL were thawed and stimulated for 12 h at 37°C with live MDCK cell–grown strain A/Sw/Eng/1353/09 (MOI of 6 for BAL and PBMCs, MOI of 0.6 for TBLN) or MDCK cell mock supernatant as control. GolgiPlug (BD Biosciences) was added according to the manufacturer’s instructions for a further 5 h before intracellular cytokine staining. Cells were stained for surface markers with CD3ε-PeCy5 PPT3 (AbCam), biotinylated CD4 clone MIL17 (in-house), with secondary streptavidin-allophycocyanin (SouthernBiotech), CD8α-FITC MIL12 (AbD Serotec), and near-IR fixable Live/Dead stain (Invitrogen). Cells were permeabilized using Cytofix/Cytoperm (BD Biosciences) as per the manufacturer’s instructions before intracellular staining with IFN-γ–PE P2G10 (AbD Serotec) and cross-reactive anti-human TNF-α–Pacific Blue Mab11 (BioLegend). Samples were fixed in 1% paraformaldehyde before analysis using an LSRFortessa instrument (BD Biosciences).

Data were analyzed using FlowJo v10 (Tree Star), and fluorescence minus one primary Ab controls were used to set gates. Samples were batch gated (Supplemental Fig. 1) on lymphocytes based on side scatter area/forward scatter area, followed by single cells on forward scatter height/forward scatter area. Live CD3^+^ cells were analyzed for expression of CD4 and CD8α. Boolean gating was used to determine the levels of IFN-γ and TNF-α expression in CD8α^hi^, CD4CD8α double-positive, and CD4 T cell subsets. Postcytometric multivariate data analysis was performed using SPICE version 5.3 (http://exon.niaid.nih.gov.).

### Statistical analysis

One-way or two-way ANOVA with a Dunnett posttest for multiple comparisons were used to compare immunized groups to the control group, and analysis was performed using GraphPad Prism 6. A general linear model (GLM), together with Tukey honestly significant difference and least significant difference post hoc tests, was used to identify any significant differences between groups for individual variables. For pen and batch, when *p* > 0.2, the variable was excluded from the GLM in a stepwise fashion. Samples taken during multiple days were analyzed using a repeated measures GLM. A nonparametric Freidman rank test was used when it was not possible to transform data to normality. A principal component analysis was performed using 17 factors (including virus titer, ELISPOT, pathology, and flow cytometry results) to explain overall variation between groups. Statistical analysis was performed using The R Project for Statistical Computing (version 3.0.1) (http://www.r-project.org/), IBM SPSS statistics software (version 21.0), and the GGobi data visualization system (version 2.1.10a) (http://www.ggobi.org/).

## Results

### Pulmonary immunization with S-FLU reduces viral load in nasal swabs and lungs

S-FLU has previously been shown to protect mice and ferrets against homologous and heterologous IAV challenge, and it has been demonstrated that to confer protection, the vaccine needs to be delivered to the lung (2, 4). We therefore administered S-FLU either i.t. or by aerosol with a nebulizer to reach the lower respiratory tract (LRT) of the pigs. We used two S-FLU vaccines: H1 S-FLU expressing the HA and NA of A/England/195/2009(pdmH1N1), and H5 S-FLU expressing the HA of the highly pathogenic avian influenza virus A/Vietnam/1203/2004(H5) and N1 from A/PR/8/34. Groups of six pigs were immunized with two doses of H1 S-FLU i.t. (H1 i.t.) or by aerosol (H1 aer) or with H5 S-FLU i.t. (H5 i.t.); the control group received medium i.t. (control). Because of the different viral titers of the H1 and H5 S-FLU vaccine stocks and different delivery routes, different doses of the viruses were administered. H5 i.t. animals received 8 × 10^7^ TCID_50_, and the H1 aer group received a lower dose of ∼6.85 × 10^6^ TCID_50_. Most likely the dose actually received was even lower owing to losses during the aerosolization and administration procedure.

**FIGURE 1.**
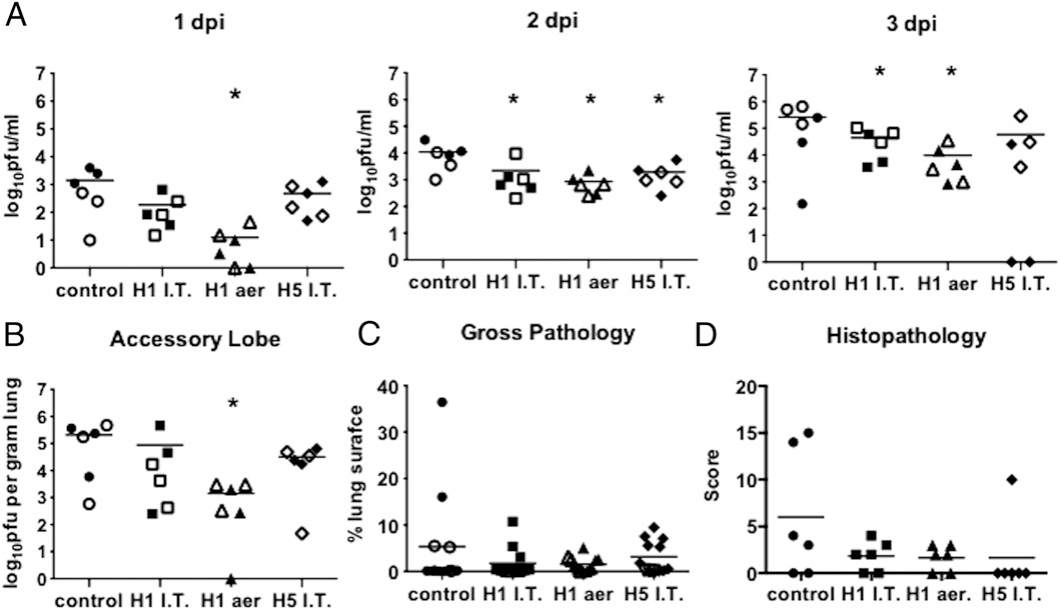
S-FLU reduces viral load in nasal swabs and lung. Pigs were immunized twice 28 d apart with either H1 S-FLU by the i.t. route (H1 i.t.), by aerosol (H1 aer), or with H5 S-FLU given i.t. (H5 i.t.). Animals were challenged with A/Sw/Eng/1353/09 at 31 (filled symbols) or 45 (open symbols) dpb. Nasal swabs were taken at 0, 1, 2, and 3 dpc and pigs were sacrificed at 3 dpc. Viral titers in the nasal swabs (**A**) and accessory lobe of the lungs (**B**) were determined by plaque assay and lungs were assessed for appearance of gross (**C**) and microscopic (**D**) pathological lesions. Each datum point represents an individual within the indicated group and bars represent the mean. *p < 0.05 versus control group determined using one-way ANOVA with a Dunnett test for multiple comparisons. I.T., i.t.

Animals were challenged with the swine pdm09 strain A/Sw/Eng/1353/09 at either 31 or 45 d after the second immunization and euthanized 3 dpc to determine the effect of immunization at the peak of virus replication. Despite immunization with >1 log less H1 S-FLU, the pigs immunized by the aerosol route showed the most efficient reduction of challenge virus in the nasal swabs at 1, 2, and 3 dpc (Fig. 1A). H1 S-FLU administered i.t. significantly reduced viral shedding at 2 and 3 dpc (*p* = 0.001), whereas H5 S-FLU reduced viral shedding at 2 dpc (*p* = 0.016) and completely prevented shedding in two individual animals at 3 dpc (Fig. 1A). Aerosol administration of H1 S-FLU also significantly reduced the viral titer in the accessory lobe of the lung (*p* = 0.006) (Fig. 1B).

No ill effects were observed in any of the pigs immunized with H1 or H5 S-FLU by either method of administration. Gross pathology after viral challenge was minimal in most immunized animals; however, the control group contained two individuals with severe gross pathology (Fig. 1C). Most lung sections had total scores of ≤4 of a maximum score of 20, showing that there were only very subtle histopathological changes. The pigs with the highest scores belonged to the control or to the H5 IT treatment groups (Fig. 1D), but no significant difference between groups was detected. In addition to the parameters scored, several lung sections showed moderate levels of hemorrhage (data not shown) that was considered most likely to be a postmortem artifact. Furthermore, collapse of alveolar spaces frequently made assessment of alveolar septal thickness difficult. However, these issues simply highlight amendments that can be considered for sample collection for future studies.

These results indicated that administration of S-FLU in pigs reduced the viral load in nasal swabs and lungs after closely matched viral challenge and that this was most efficiently induced by aerosol administration. H5 S-FLU reduced the viral load in nasal swabs and lung following heterologous viral challenge, but this was significant in nasal swabs only at 2 dpc.

### Influenza-specific immune responses in PBMCs

As the analysis of samples from pigs challenged at days 31 and 45 did not reveal any significant differences (Fig. 1), for simplicity in presentation the results of the immunological assays carried out on pigs challenged on both days have been amalgamated.

Because most commercial vaccines used to control influenza virus elicit a strong Ab response in the host, we first determined whether the reduced viral replication after S-FLU immunization resulted from a humoral immune response. HAI and microneutralization titers in serum and BAL fluid of all immunized groups were comparable with unimmunized controls at 3 dpc (Table I), consistent with previous studies showing lack of neutralizing Ab responses following S-FLU immunization of mice and ferrets (2, 4). Influenza-specific IgG and IgA were also quantified by ELISA in BAL fluid samples at 3 dpc and similarly very low endpoint titers for IgG and IgA were detected (Table I).

**Table I.** Serum and BAL fluid A/swine/1353/09(pdmH1N1)–specific HAI, MN, IgG, and IgA titers

Group	Serum				BAL Fluid		BAL Fluid 3 dpc	
	HAI*^a^*		MN*^b^*		HAI*^a^*	MN*^b^*	ELISA*^c^*	
	0 dpc	3 dpc	13 dpb	3 dpc	(3 dpc)	(3 dpc)	IgG	IgA
Control	8.0 ± 0.0	7.3 ± 1.5	<10	<10	4.0 ± 0.0	<10	0.8 ± 1.1	0.0 ± 0.0
H1 i.t.	9.6 ± 3.2	14.7 ± 8.5	<10	<10	5.3 ± 1.9	<10	3.6 ± 7.0	2.4 ± 2.2
H1 aer	11.2 ± 4.0	14.7 ± 3.0	<10	<10	5.6 ± 2.0	<10	3.0 ± 7.0	2.0 ± 2.0
H5 i.t.	8.0 ± 0.0	10.0 ± 4.5	<10	<10	12.0 ± 4.0	<10	8.0 ± 8.8	2.0 ± 1.3

^*a*^ HAI titers were determined in serum at 0 and 3 dpc and BALF after sacrifice at 3 dpc using 4 HA units of A/Sw/Eng/1353/09 virus. Results shown are the mean of six animals ± SD. As a positive control, serum from pigs immunized with commercial A(H1N1)pdm09 vaccine Pandemrix (GlasxoSmithKline) and challenged with A/England/195/09(pdmH1N1) virus as described (6) was used and gave a titer of 2048.

^*b*^ MN titers were determined in serum at 13 dpb and 3 dpc and BALF after sacrifice at 3 dpc using 100 PFU A/Sw/Eng/1353/09 virus. Results shown are the mean of six animals. Serum and BALF collected from A/Sw/Eng/1353/09 virus–infected pigs at 14 dpc were used as controls with both giving a titer of 80.

^*c*^ IgG and IgA titers in BALF after sacrifice at 3 dpc. Results shown are the mean of six pigs ± SD. As a positive control, BALF collected from A/Sw/Eng/1353/09 virus–infected pigs at 14 dpc was used and gave an endpoint titer of 1024 for IgA and 128 for IgG.

**FIGURE 2.**
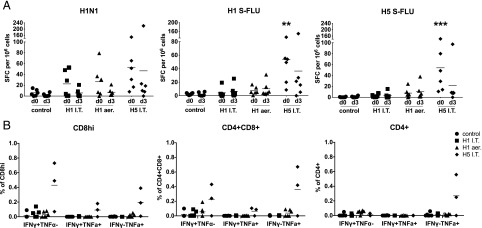
Cytokine responses in PBMCs following S-FLU immunization. Pigs were immunized twice 28 d apart with either H1 S-FLU by the i.t. route (H1 i.t.), by aerosol (H1 aer), or with H5 S-FLU given i.t (H5 i.t.). Animals were challenged with A/Sw/Eng/1353/09 and PBMCs isolated at 0 and 3 dpc. (**A**) Numbers of IFN-γ SFC were determined by ELISPOT following stimulation with the challenge virus, H1 S-FLU, or H5 S-FLU. (**B**) Flow cytometry was used to quantitate the frequency of IFN-γ– and TNF-α–producing cells within CD8^hi^, CD4^+^CD8^+^, and CD4^+^ after stimulation with A/Sw/Eng/1353/09. Each datum point represents an individual within the indicated group. Results are representative of two independent experiments. **p < 0.005, ***p < 0.0005 versus control group determined using two-way ANOVA with a Dunnett test for multiple comparisons. I.T., i.t.

We next determined average influenza-specific T cell responses in PBMCs by IFN-γ ELISPOT at 0 and 3 dpc. All S-FLU–immunized groups showed a virus-specific response to the challenge pdmH1N1 virus of ∼30–60 spot-forming cells (SFC) per 10^6^ cells just before the challenge (0 dpc), indicating that all animals had been immunized successfully. H5 i.t. immunized animals, which showed the strongest T cell response to A/Sw/Eng/1353/09 (*p* = 0.001), responded equally well to stimulation in vitro with H1 S-FLU or H5 S-FLU vaccine constructs, whereas H1 i.t. and H1 aer animals made minimal responses to H1 S-FLU or H5 S-FLU (Fig. 2A). At 3 dpc the number of SFC following pdmH1N1 stimulation declined in all immunized groups, most likely due to the migration of influenza-specific PBMCs to the lung. Intracellular cytokine staining of PBMCs at 3 dpc shows the highest IFN-γ and TNF-α responses in the H5 i.t. group (Fig. 2B).

Taken together, these data show that the magnitude of the response to IAV in peripheral blood, measured either as number of IFN-γ□ or TNF-secreting cells, does not correlate with reduction of viral load in nasal swabs or the accessory lung lobe. It is not clear why PBMCs from the H1 i.t. and H1 aer–immunized animals do not respond in vitro to stimulation with H1 S-FLU or H5 S-FLU, whereas H5 i.t. immunized animals do, but this may be related to the higher dose of H5 S-FLU administered.

### BAL and TBLN influenza-specific responses

**FIGURE 3.**
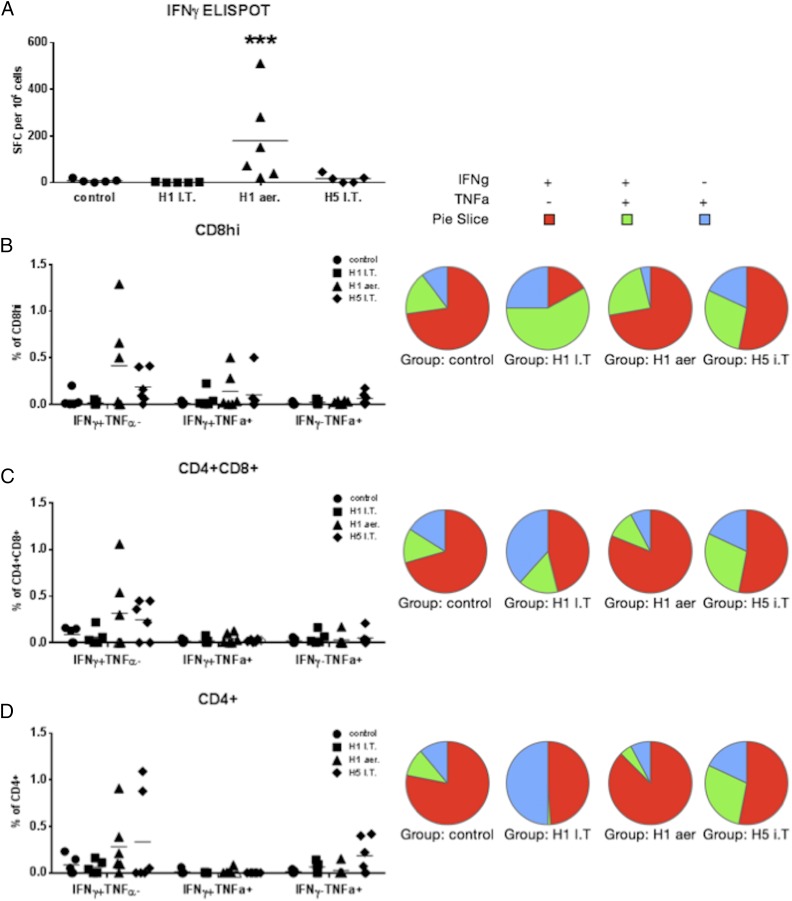
S-FLU immunization induces influenza-specific T cell responses in the BAL. Pigs were immunized twice 28 d apart with either H1 S-FLU i.t. (H1 i.t.), by aerosol (H1 aer), or with H5 S-FLU i.t. (H5 i.t.). Animals were challenged with A/Sw/Eng/1353/09 and sacrificed at 3 dpc. Cells were stimulated with the challenge virus. (**A**) Numbers of IFN-γ–secreting cells were determined by ELISPOT in the BAL 3 dpc. Flow cytometry was used to quantitate the frequency of IFN-γ– and TNF-α–producing cells (graphs) and the qualitative bifunctional cytokine response (pie charts) within CD8^hi^ (**B**), CD4^+^CD8^+^ (**C**), and CD4^+^ (D). Each datum point represents an individual within the indicated group. Results are representative of two independent experiments. ***p = 0.001 versus control group determined using two-way ANOVA with a Dunnett test for multiple comparisons. I.T., i.t.

We next measured the local influenza-specific immune responses in cells from BAL and TBLN, the main draining lymph nodes of the lungs. H1 aer immunization induced the highest numbers of IFN-γ–secreting cells in the BAL (∼200 SFC per 10^6^ cells, *p* = 0.001), whereas a much lower response occurred in the H5 i.t. immunized animals and none in the H1 i.t. and control groups (Fig. 3A).

Because it has been suggested that cells simultaneously producing IFN-γ, IL-2, and TNF-α may provide optimal effector function and protection against viral infections (7), we defined the proportions of bifunctional cells in our immunized animals. We performed intracellular staining for IFN-γ and TNF-α on BAL cells stimulated with A/Sw/Eng/1353/09. H1 aer immunization induced the strongest, single IFN-γ responses by CD8 and CD4CD8 cells (0.41% in CD8 and 0.32% in CD4CD8 cells), followed by the H5 i.t. regimen (Fig. 3B, 3C). The proportion of cells producing TNF-α was very low in all cell subsets, and neither TNF-α single-producing nor IFN-γTNF-α double-producing cells differed between the groups (Fig. 3B–D). The pie charts summarize the proportions of single and double cytokine-producing cells in the BAL. The H1 aer and H5 i.t. regimes appear to induce different proportions of single or double cytokine-producing influenza-specific cells compared with H1 i.t. and controls. However, H1 i.t. and controls induced very few Ag-specific cells so that interpretation of these data requires caution. Nevertheless, H1 aer immunization induced greater numbers of IFN-γ single producers in all cell subsets.

**FIGURE 4.**
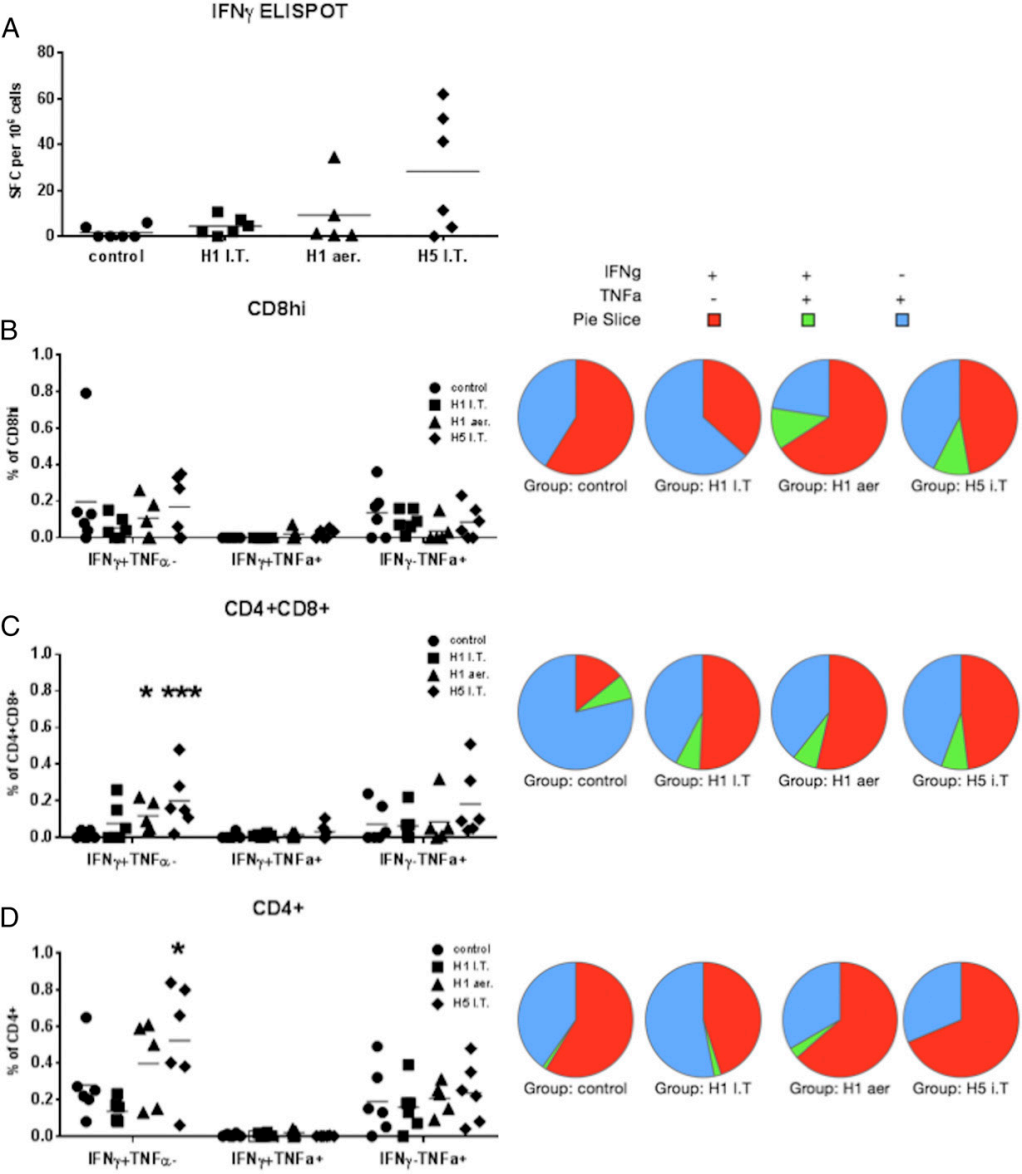
Cytokine responses in TBLN to influenza virus. Pigs were immunized twice 28 d apart with either H1 S-FLU i.t. (H1 i.t.) or by aerosol (H1 aer) or H5 S-FLU i.t (H5 i.t.). Animals were challenged with A/Sw/Eng/1353/09. (**A**) Numbers of IFN-γ–secreting cells in the TBLN at 3 dpc were determined by ELISPOT. Flow cytometry was used to quantitate the frequency of IFN-γ^+^ and TNF-α^+^ cells (graphs) and the qualitative bifunctional cytokine response (pie charts) within CD8^hi^ (**B**), CD4^+^CD8^+^ (**C**), and CD4^+^ (**D**). Each datum point represents an individual within the indicated group. Results are representative of two independent experiments. *p = 0.05, **p = 0.0085 versus control group determined using two-way ANOVA with a Dunnett test for multiple comparisons. I.T., i.t.

TBLN responses differ from those in the BAL. H5 i.t. immunization induced the strongest IFN-γ ELISPOT response followed by the H1 aer regimen (Fig. 4A). Similarly, the H5 i.t. regimen induced the significantly highest CD4CD8 IFN-γ–producing cells as determined by intracellular cytokine staining staining (*p* = 0.009) followed by the H1 aer regimen (*p* = 0.047) (Fig. 4C). TNF-α production was higher compared with the BAL, although no differences between the groups were detected in the proportion of single TNF-α– or double TNF-αIFN-γ–producing cells. As for BAL, the pie charts show that the best protected H1 aer group had the highest proportion of IFN-γ single-producing cells (Figs. 4B–D).

In summary, the most protective H1 aer immunization induces a strong BAL and weaker TBLN response to IAV. These responses consist of cells with high amounts of intracellular IFN-γ, which correlate with and may account for their protective efficacy.

### S-FLU may have the potential to be a candidate universal vaccine

**FIGURE 5.**
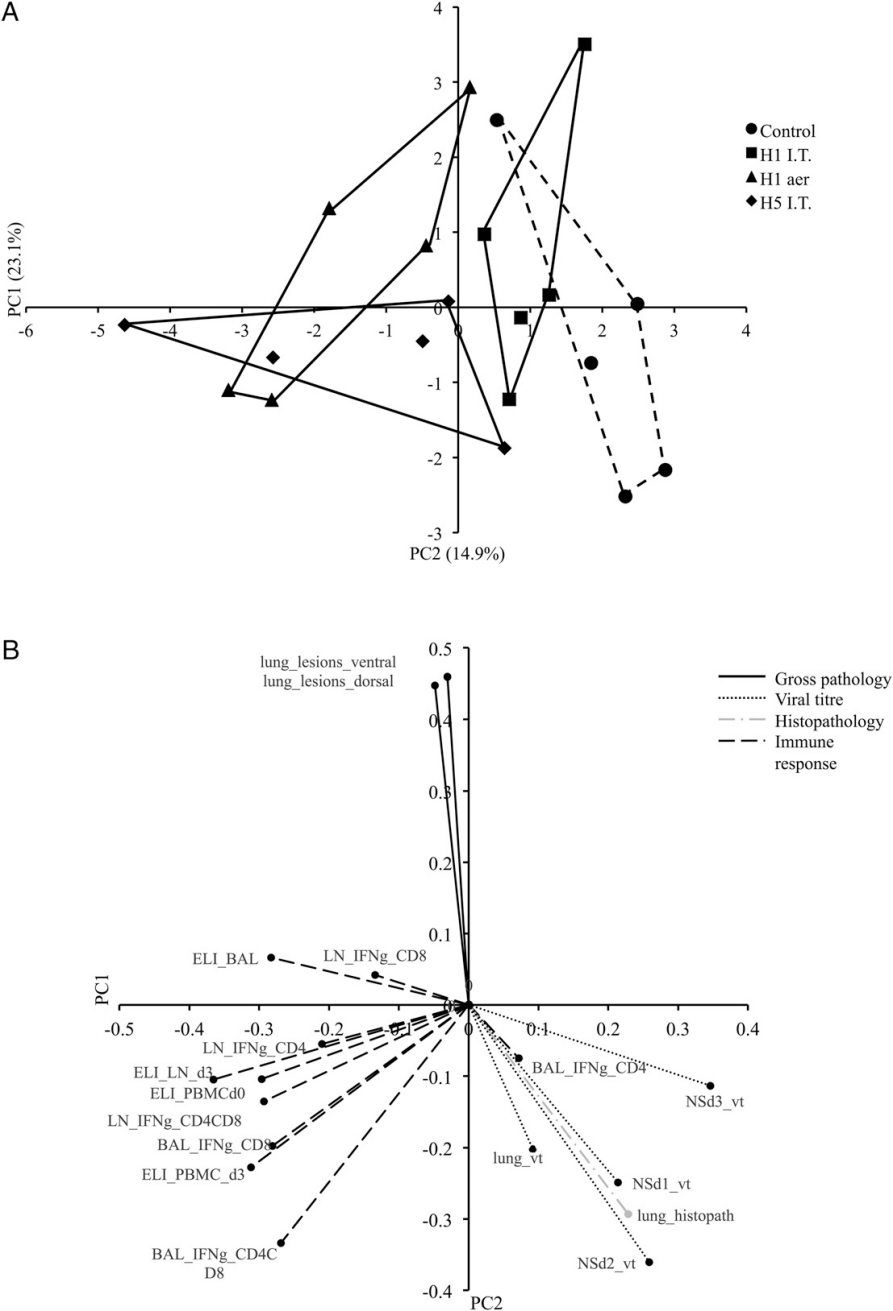
(**A**) PCA plot of response to swine influenza infection. PC1 explained 23.1% of the total variation between pigs and PC2 explained a further 14.9% of the variation. Treatment groups are represented by different symbols and each point represents an individual pig. (**B**) PCA loadings for each individual input variable. Three distinct clusters can be observed comprised of gross pathology, virus load and histopathology, and immune response. d, days postchallenge; ELI, ELISPOT; I.T., i.t.; LN, lymph node; NS, nasal swab; vt, viral titer.

Principal component analysis (PCA) was used to reduce a large number of original variables to a smaller number of undefined, underlying variables that are responsible for the variation in the data. The first principal component, PC1, could explain 23.1% of the total variation between pigs (Fig. 5A). A general linear model showed that PC1 was significantly associated with immunization status (*p* = 0.0014). In particular, the post hoc test revealed significant differences between the control and most of the immunization groups (H1 aer, *p* = 0.0009**;** H5 i.t., *p* = 0.0012). A second component, PC2, explained a further 14.9% of the variation and could be theorized to be associated with route of administration, although there was no significant difference between the aerosol and i.t. delivery routes (data not shown). Because of missing data points, it was not possible to obtain a full set of principal components for four pigs (one in each treatment group).

The variables used for the PCA were assigned to one of four groups relating to gross pathology, viral titer, histopathology, or immune response. Fig. 5B shows the PCA loadings for each of these variables. The loadings explain how much variation within a particular variable can be assigned to a principal component. Three distinct clusters can be identified; viral titer and histopathology cluster together in the same quadrant, suggesting that there is an association with viral load and histopathological signs. Interestingly, IFN-γ production by CD4^+^ T cells in BAL also appears in this quadrant. Although gross pathology and histopathology appear in different quadrants, they do not directly oppose each other, suggesting that there is still some level of association between the two variables.

Considering Fig. 5A and 5B together, the PCA analysis shows that virus titer and histopathology scores were highest in the control group and lowest in the H1 aer group. Note that the H5 i.t. group also had a reduction in viral load and histopathology. Additionally, the figures show that the strongest immune response was seen in the H5 i.t. group, closely followed by the H1 aer group. This may be explained by the heterologous challenge virus used in the H5 i.t. group, as opposed to homologous challenge. The H1 i.t. and H1 aer groups also had lower gross pathology scores than did the control and H5 i.t. groups. Consistent with the previous analyses on individual variables, it appears that the H1 aer vaccination was most effective at reducing viral load and histopathological and gross pathological signs, as well as inducing an effective immune response to challenge with a homologous virus.

## Discussion

Abundant evidence in humans and animals shows that natural influenza virus infection or immunization with live attenuated influenza viruses can induce a degree of cross protection (1, 8, 9). Cross-reactive CD4 and CD8 T cells or anti-HA stem Abs have been postulated as correlates of this cross protection in humans, but vaccines currently in use induce these responses poorly. In the present study, we have used a novel candidate universal vaccine, S-FLU, based on the suppression of HA signal sequence. S-FLU has the advantage of inducing an immune response to all viral components, but without replication of virus or danger of reassortment of HA viral RNA into seasonal influenza, making immunization directly to the site of infection safe.

Our data show that immunization with S-FLU–expressing H1 HA (H1 S-FLU) reduces the viral load in nasal swabs and lungs after challenge with the closely matched pdmH1N1 virus strain. The reduction of viral load was greatest after aerosol administration of S-FLU. Detectable neutralizing Ab was not produced in S-FLU–immunized pigs. Rather, the reduction of viral load in the H1 aer group correlated with the presence of IFN-γ–producing CD8 or CD4CD8 double-positive cells in the BAL. CD4CD8 double-positive cells in pigs are activated memory CD4 cells (10), meaning S-FLU appears capable of inducing both CD4 and CD8 memory, both of which have been shown to be important in protective immunity to influenza (11–13). H5 i.t. immunization induces a much weaker BAL response, even though a higher dose of virus was administered, but stronger TBLN and PBMC responses. These results suggest that the effects of priming by aerosol and i.t. administered vaccines differ. The higher TBLN response and lower BAL response after i.t. immunization suggest either that priming by this route may have slower kinetics than aerosol immunization so that responding cells are still in the draining nodes at the time of euthanasia or that cells primed by this route do not acquire homing molecules allowing them to enter the alveolar spaces. It is also possible that the HA and NA molecules expressed on different S-FLUs may influence which cells within the respiratory tract can be infected. This in turn might influence the magnitude and nature of the immune response. Further experiments are required to define more clearly distribution of S-FLU delivered to pigs by aerosol or to the upper trachea and the properties of the Ag-specific cells induced. Our preliminary data (not shown) indicate that S-FLU administered intranasally did not induce lung immune responses or reduce viral load. Finally, we have recently shown that S-FLU can induce inhibitory Abs to the encoded NA in mice (T. Powell, P. Rijal, K.-Y. Huang, R. McEwen-Smith, H. Byun, M. Hardwick, L. Schimanski, R. Daniels, and A. Townsend, submitted for publication), so it is possible that the homologous immunization with H1N1 S-FLU induced a protective anti-N1(Eng) response. This is less likely with the H5N1 S-FLU, which encoded a distantly related N1 sequence from A/PR/8/34.

Recent overwhelming evidence shows that immunization or infection via the respiratory tract results in generation of lung-resident memory T cells, which are much more numerous than previously thought and are critical for protection (14–16). These lung-resident T cells are phenotypically different from their peripheral blood counterparts and respond more vigorously to respiratory viruses, thus providing additional protection against infection. Further investigation will be needed to reveal the distribution within the lungs of memory cells induced by different methods of immunization and determine whether the BAL cells we detect in H1 aer–immunized pigs are part of the lung-resident memory population.

The influenza-specific responses in our S-FLU–immunized animals were detectable up to 45 dpb but neither the frequency of IFN-γ–secreting cells nor the presence of multifunctional cells in the blood correlated with reduction of viral burden in nasal swabs or lungs. In humans, specific subsets of blood memory T cells with specificity for conserved internal proteins of influenza virus have been associated with protection against infection (11–13). Additional work may indicate whether such memory subsets exist in pigs and are induced by immunization with S-FLU. Influenza-specific cross-reactive CD8 T cells are also detectable in the lungs and persist for at least a year in mice and humans (14, 17). Longer studies in pigs will be required to establish the persistence of memory in this species and whether lung immune responses correlate with protective immunity during an extended period.

Our experiments in pigs demonstrate that targeting the LRT by aerosol is an efficient way to induce an immune response and to reduce viral load. This is in agreement with earlier studies showing that for optimum induction of protective T cell immune response in mice and ferrets, virus infection of the lung is required, as opposed to infection of the upper respiratory tract or other peripheral sites (18–20). Aerosol delivery of H1 S-FLU (at least 4-fold less than H1 S-FLU i.t. and ∼1 log less than H5 S-FLU i.t. administered dose) induced a potent immune response in the BAL compared with the i.t. route of administration and was the most efficient in reducing viral load. A higher dose H5 i.t. immunization provided some degree of reduction of viral load after heterologous pdmH1N1 challenge. However, our method of i.t. delivery to the upper trachea clearly fails to immunize the lung efficiently and therefore further studies using aerosol administration will be required to establish whether S-FLU provides a high degree of heterologous as well as homologous protection in pigs. Similar comparisons between i.t. and aerosol delivery have been performed with adenoviral-vectored tuberculosis or Ebola vaccines in nonhuman primates, and in both cases aerosol delivery offered superior protection compared with other mucosal routes (21, 22).

Although aerosol delivery of measles vaccine has been successfully deployed in humans (23, 24), to our knowledge this is the first report of delivery of an influenza vaccine to pigs by aerosol. Feng et al. (25) delivered a *Mycoplasma hyopneumoniae* vaccine by aerosol to pigs, and although they showed that the vaccine reached the LRT and induced local IgA, they did not assess protection or T cell responses induced by the immunization. Further studies will be required to develop practical devices for aerosol immunization in the field, but our data provide proof of principle that S-FLU can be efficiently delivered by aerosol to a large animal, strongly suggesting that this would be a highly efficient method of immunizing both pigs and humans against influenza viruses.

Various live attenuated influenza vaccines (LAIV) have been developed and shown to be effective when delivered to pigs by the intranasal route (26–29). Loving et al. (29) used LAIV temperature-sensitive polymerase mutants to show prevention of viral shedding and transmission, but this did not correlate with Ab titers in serum or mucosal secretions. At first sight this result appears to contradict our finding that delivery of the vaccine to the LRT is the most efficient means of inducing a protective immune response. There are also other differences between the present study and that of Loving et al. (29). They used much younger (5 wk) and smaller animals and a different challenge virus (H3N2), and the details of the method of intranasal administration are not clear. Furthermore, no assays of T cell immunity were carried out so that it is not possible to exclude the possibility that a lung immune response was induced. Only head-to-head studies of different vaccines, together with assays of local and systemic immunity, will reveal the factors underlying these apparent differences.

Vaccine-associated enhanced respiratory disease (VAERD) has been reported after immunization with inactivated swine H1N2 vaccine followed by infection with pdmH1N1 and correlated with the presence of anti-HA stem Ab (30). However, it has been demonstrated that VAERD could not be induced after administration of LAIV (5). This and our data showing lack of VAERD after S-FLU immunization support the idea that mucosal administration of vaccines avoids this complication.

The licensed cold-adapted LAIV (FluMist) can induce cross-protective immunity when delivered to the lung (2, 4, 31), but not the upper respiratory tract of mice (20). However, there are two barriers to delivering existing LAIV to the LRT. The first is that delivery to the LRT of humans, although it may be the most effective means to induce protective immunity, may be dangerous, as LAIV retains some potential to replicate. Second, the viral RNA encoding pandemic HA in the vaccine virus is capable of reassortment with circulating seasonal viruses, with the potential to render the latter highly virulent (32). In contrast, S-FLU should be safe for aerosol administration to the lung because it cannot replicate, nor can it donate, a viable HA gene to seasonal influenza.

Although understanding of immunity to influenza virus remains incomplete, our experiments suggest an important role for local lung immunity. Heterotypic immunity of the type induced by S-FLU, which is predominantly T cell mediated, does not prevent viral entry, but limits viral replication. Additional studies will be required to test whether the reduction in viral load shown in these studies is sufficient to block transmission and prevent disease. Nevertheless, the best protected lungs showed the highest numbers of Ag-specific cells in the BAL, indicating that local immunity in the lung is an important mechanism by which universal vaccines can confer protection. These data therefore have important implications for the design of pig and human universal vaccines against influenza. Challenges for the future will include the design of immunization regimes and devices that can safely induce long-lasting lung-resident memory in humans and livestock.

## Supplementary Material

Data Supplement
