# Supplementary material for: Aerosol Delivery of a Candidate Universal Influenza Vaccine Reduces Viral Load in Pigs Challenged with Pandemic H1N1 Virus
Source: J Immunol. 2016 May 6;196(12):5014–23. doi: 10.4049/jimmunol.1502632 (PMC4891568; doi:10.4049/jimmunol.1502632)
Supplement: Data Supplement [file JI_1502632.zip › JI_1502632_Supplemental_Material_1.pdf]

Supplemental Table. Parameters used for scoring of microscopic histopathology lesions.

Score	0 (None)	1 (Minimal)	2 (Mild)	3 (Moderate)	4 (Severe)
Airway epithelial necrosis, attenuation or disruption	None	Rare foci affecting 1-2 airways	Affecting more than 2 airways and up to one third of airways	Affecting more than one third and up to two thirds of airways	Affecting more than two thirds of airways
Airway inflammation	No inflammation	Sparsely scattered granulocytic inflammatory cells affecting occasional airways	More than a few scattered neutrophils or eosinophils (e.g. intraluminal aggregation(s)) affecting up to one third of airways	Inflammation as score 2 affecting more than one third, and up to two thirds, of airways	Inflammation as score 2 affecting more than two thirds of airways
Peribronchiolar and perivascular lymphocytic cuffing	No discernible peribronchiolar / perivascular cuffing	Occasional incomplete, or loosely formed, cuffs or lymphocytic aggregations	Numerous cuffs, predominantly incomplete and loosely-formed with lesser well-formed complete cuffs	Numerous cuffs, approximately half or more well-formed, and may have a few broad, dense cuffs	Numerous cuffs, predominantly well-formed with numerous broad, dense cuffs
Alveolar cellular exudate/oedema and interlobular oedema	None	Occasional alveoli affected – eosinophilic fluid	Confluent alveoli and/or interlobular septal involvement affecting up to one third of lung	Confluent alveoli and/or interlobular septal involvement affecting more than one third and up to two thirds of lung	Confluent alveoli and/or interlobular septal involvement affecting more than two thirds of lung
Alveolar septal inflammatory cells and cellularity	Septae typically 1-2, or occasionally 3, nucleated cells wide and absence of inflammatory cells	As score 1 but with scattered inflammatory cells within alveolar walls - often granulocytes most likely within blood vessels	Focal or multifocal alveolar septal inflammation with regions of mild thickening of septae and increased mononuclear cells (affected septae 3-4 nuclei wide) or may have scattered increased type II pneumocytes	Focal or multifocal septal inflammation with regions of moderate thickening of septae and increased mononuclear cells (affected septae 5 or more nuclei wide). May have increased type II pneumocytes	Coalescing to diffuse alveolar septal inflammation (septae 3 or more cells wide)

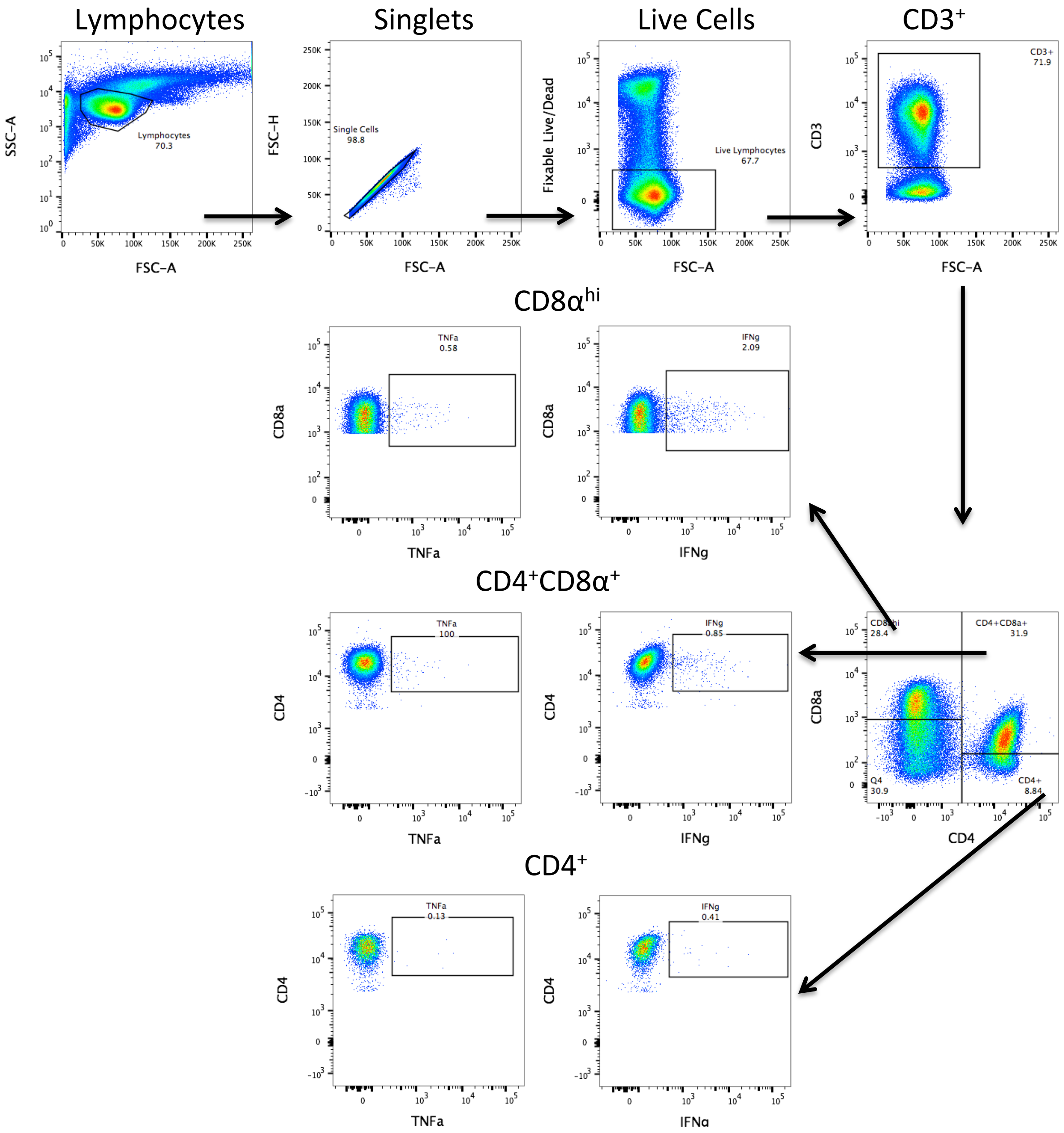

Supplemental Figure. Gating strategy for analysis of intracellular cytokine responses. Lymphocytes based on SSCA/FSCA, followed by single cells on FSCH/FSCA. Live CD3 positive cells were analysed for expression of CD4 and CD8α. Boolean gating was used to determine the levels of IFNγ and TNFα expression in CD8α high, CD4CD8α double positive and CD4 T cell subsets.
